# Periodontal disease and its impact on general health in Latin America – Diagnosis: Latin America and the Caribbean Consensus 2024

**DOI:** 10.1590/1807-3107bor-2024.vol38.0119

**Published:** 2024-11-22

**Authors:** Cassiano Kuchenbecker RÖSING, Juliano CAVAGNI, Gerson Pedro José LANGA, Willy BUSTILLOS TORREZ, Juan Antonio CEPEDA BRAVO

**Affiliations:** (a) Universidade Federal do Rio Grande do Sul – UFRGS, School of Dentistry, Department of Periodontology, Porto Alegre, RS, Brazil.; (b) Instituto Superior de Ciência e Tecnologia de Moçambique – ISCTEM, Maputo, Mozambique; (c) Universidad Privada Franz Tamayo (UNIFRANZ), Research department, Bolivia.; (d) Universidad Autónoma de San Luis Potosí, Facultad de Estomatología, Department of Periodontology, San Luis Potosí, Mexico.

**Keywords:** Diagnosis, Periodontal Diseases, Latin America

## Abstract

This is a new version of the LAOHA Consensus on Periodontal Disease and its impact on general health in Latin America. Five years after the first version, knowledge has increased, and diagnosis of periodontal disease has evolved. Of especial interest relative to this topic has been the emergence of studies that have used the AAP/EFP Classification of Periodontal and Peri-Implant Diseases and Conditions since it is the core (?)of the art of diagnosis in preventive and therapeutic strategies. To make an accurate and effective diagnosis, knowledge about the health-disease process is fundamental. This article updates and demonstrates challenges in periodontal diagnosis, especially in Latin American countries. Considering that periodontal diagnosis should be based on knowledge of the etiopathogenesis of periodontal diseases, this article points out aspects developed in the last few years and emphasizes the knowledge that has been established, considering diagnosis of periodontal diseases. The evidence available stresses the importance of interviewing the patient, by implementing periodontal charting, and requesting any imaging and other complementary tests necessary. An important observation is that the partial periodontal data recordings used for screening (up to now) are not diagnostic methods and might underestimate disease. Moreover, in this article approaches to other forms of recognizing periodontal diseases are described, which could be used, however, not for the purpose of diagnosis, but for increasing awareness and eventually for referring individuals. In Latin American countries there is a need to increase the awareness of periodontal diseases among both the population and the profession, with particular emphasis on prioritizing correct periodontal diagnosis. In Dental education, strategies need to be established with the purpose of understanding that diagnosis is pivotal to any clinical approach.

## Introduction

In 2019, the Latin American Oral Health Association held a Consensus Meeting with representatives of several countries and representative Scientific Associations, which generated important publications for the region, in an endeavor to address the impact of periodontal diseases in Latin America. After 5 years, a new Consensus is warranted, since knowledge has evolved considerably. The section about diagnosis, which is of interest to the present article, was published by Rösing et al.,^1^ after the participants in the Consensus Meeting had made their contributions, The present article updates and offers new insights into the previously published Consensus Report. It is a narrative review, performed after an extensive and systematic search related to periodontal diagnosis.

Diagnosis of periodontal diseases and conditions has been the subject of a series of controversies that led to difficulties in communication, and especially related to different clinical approaches in Dentistry. In the Glossary of Periodontal Terms of the American Academy of Periodontology, periodontal diagnosis has been defined as “The process (or opinion derived from the process) of identifying the nature and cause of a disease of the periodontium; relevant information used in this process typically includes medical and dental histories, clinical and radiographic examination of the patient, and laboratory findings”.^2^ The art of diagnosis should be considered above any classification system which, per se, is an arbitrary way of distinguishing different forms of disease and conditions. However, classification systems might shed light on the possibility of communication. This article approaches the diagnosis of the periodontal health/disease process, in an endeavor to understand its challenges, and to propose possible solutions, especially for Latin American countries. The article will be public ally evaluated before its final version and contributions will be included in the article.

Historically, Dentistry has focused mainly on dental caries since this is still the major cause of tooth loss, pain, and impairment of oral health. This focus led to underdiagnosis of periodontal diseases and other oral conditions. The decline in estimates of dental caries occurrence, and understanding of the importance of more comprehensive oral care, has led to the understanding that periodontal diseases need to be looked upon with more attention, from a health perspective of both individuals and populations. This is supported by evidence of the role of periodontal diseases in oral outcomes e.g. tooth loss, as well as possible relations with other systemic conditions and oral health-related quality of life.^3-5^ Studies have demonstrated that routine periodontal diagnosis has not been performed as would be expected. This is probably related to the specificities in periodontal diagnosis as well as a reflection of dental education still mainly focused on dental caries and its consequences.^4,6^Furthermore, an important fact is that health systems do not value periodontal diagnosis and treatment as they should.

### Diagnosis of the periodontal health/disease process

It is important to recognize that diagnosis of the periodontal health/disease process differs completely in epidemiological and clinical settings. Epidemiological surveys describe the occurrence of states of health and disease in populations, associating them with possible risk factors/indicators. In this sense, epidemiological studies are not focused on individual diagnosis. Moreover, notably epidemiological studies use cut-off points that are not always the outcomes most used in clinical settings. There is a misunderstanding about the role of epidemiological studies that leads to misinterpretation of periodontal disease diagnoses. Outcomes in epidemiological surveys are arbitrarily posed especially related to the main objectives of the study. Several surveys have been reported in different articles with distinct primary outcome cut-off points. Data from epidemiological studies will be used to build the knowledge that will guide the process of individual diagnosis.^7,8^

As stated in the definition of periodontal diagnosis, from an individual perspective, it should be focused on the person as a whole, combined with information not only derived from the clinical examination.^2^ For this reason, the vision of an individual diagnosis based on local and systemic conditions and risk factors of each patient must be an exercise that leads to the best treatment strategy. Moreover, there is one point of consensus: periodontal disease cannot be diagnosed after the tooth is about to be lost due to periodontal breakdown! This is lack of responsibility of the professional(?), who seems to underdiagnose periodontal diseases.

Periodontal diseases have been classified in different ways. The point that needs to be reinforced is that the periodontal health/disease process clinically manifests in two main types of impairment: gingivitis and periodontitis. The distinction between these two diseases is mainly based on the concomitant occurrence of loss of attachment. Gingivitis is an inflammatory process triggered by the presence of supragingival biofilm and is not associated with loss of the periodontal apparatus. Periodontitis occurs after imbalance between the presence of subgingival biofilm and the host response, leading to loss of periodontal attachment and bone. It is well recognized that in its causal chain, periodontitis has important risk factors that should be emphasized in prevention, diagnosis and treatment. Since both diseases have a background of an inflammatory process, diagnosis should include these aspects in the interview with the patient, in the physical examination, and with additional diagnostic tests that could help in the diagnosis.^9,10^

In 2018, a Joint Workshop hosted by both the European Federation of Periodontology and the American Academy of Periodontology launched a new classification system for periodontal and peri-implant diseases and conditions. An impressive effort was made to improve the existing classification systems.^10^ Professionals usually require a learning curve to enable the new classification system to be adopted worldwide. The system comprises gingival health, gingivitis, periodontitis and peri-implant diseases and conditions. After more than 5 years, the dental profession has used the system and continues in the learning curve.^8^ But it should be understood that the AAP/EFP system is no longer new and is suffices for an evidence-based diagnosis of periodontal diseases.^7^ The system is based on the best available evidence, however, in some situations low-level evidence had to be used. An extremely important aspect to understand is that the classification system was not meant to be a priority for epidemiology or research, but was meant for individual diagnosis. Of course, it needs to be understood that the extensive study conducted in the literature should be the basis for epidemiological and research purposes, without the need for complete standardization between these two activities. This article acknowledges that a part of the system was dedicated to defining gingival health - from pristine gingival health to clinically healthy gingiva. In addition, this article points out that periodontitis was mainly classified into stages and grades. This system allows the understanding that in each patient, both rate of progression and the way the function is affected, accounts for tooth loss, for example.^10^

It is also of interest to mention that in 2019 an important paper was published alerting dental professionals about the importance of ending the neglect of global oral health, and suggesting radical action. This included comprehension of the broad spectrum of pathogenesis of the disease process and, of course, devising an amplified strategy for diagnosis and prevention.^11^

### Periodontal diagnosis in practice

This article emphasizes the importance of general practitioners and specialists being well trained in diagnostic capabilities. Specialists should also dedicate time to more in depth examination of complex cases.^7^ Furthermore, although this article has focused on periodontal diagnosis, it is mandatory for dentists to be proficient in oral health diagnosis. For example, root caries is a very frequent situation in periodontal individuals, and it should not be underdiagnosed. It should be borne in mind that before being a specialist in any area of Dentistry, professionals are general dentists and therefore, diagnosis should not be considered part of specialized care.^12^

The interview with the patient is of utmost importance in periodontal diagnosis. This is a challenge to the professional, since a comprehensive interview is one of the keys of diagnosis of all conditions, including periodontal diseases. It is noteworthy that simply reading even validated questionnaires might not be enough to collect data from a suffering individual In terms of other systemic conditions since over 50 conditions have been associated with the occurrence of periodontal diseases, ranging from hormonal changes, exposure to environmental factors through to rare syndromes.^9,10^ Professionals need to have this knowledge and incorporate it into the interview with the patient. Moreover, since periodontal diseases are linked to behavioral components including oral hygiene methods, these should be part of the diagnostic process. The interview with the patient is also part of the treatment. For example, motivational interview strategies are used both in diagnosis as in clinical management of chronic diseases.^13-15^

The physical examination should consider the understanding that periodontal diseases are of a chronic nature. The progression of untreated periodontitis is known to be slow, therefore limiting rapid clinical impact.^16^ In this sense, periodontal physical examination still is based on the history of disease. In addition, the presence of inflammatory signs is of utmost importance in diagnosis of periodontal diseases. Therefore, the diagnostic tool most used is periodontal probing, for the purpose of understanding both the inflammatory status (e.g. with probing depth or bleeding on probing) or the history of disease (with loss of attachment). This is also one of the best tools for monitoring progression of disease over time.^17^ Considering the foregoing information, it is a consensus that in some way, every dentist needs to perform periodontal probing in every patient. This is one of the challenges of periodontal diagnosis, since there is a perverse understanding that periodontal diagnosis is for specialists.^1^

The AAP/EFP classification system calls for probing attachment loss to enable better diagnosis of periodontitis.^10^ Of course, for epidemiological reasons, periodontal probing to obtain the history of disease progression should be performed mainly in adult individuals. Children should be periodontally diagnosed with probing and/or radiographs if they have family history of periodontal disease. On the other hand, measurements for detection of gingival inflammation are needed from childhood.^18,19^

Periodontal probing is known to be time consuming and laborious and this is one of the reasons why it has not been as widely used as expected. This article urges that dental training must reinforce the importance of using this tool to increase the quality of oral diagnosis.^10^ Reports have been observed about different types of probes (manual vs. automatized/computerized).^17^ Therefore, practitioners are encouraged to use any type of probe. The gold standard for periodontal diagnosis is full mouth periodontal examination, i.e. periodontal probing in six sites per tooth (disto-buccal, mid-buccal, mesio-buccal, disto-palatal/lingual, mid-palatal/lingual, mesio-palatal/lingual). When this approach is adopted, there are few chances of misdiagnosis of periodontal diseases. ^20,21^ However, other simplified approaches have been proposed in an attempt to increase the number of dentists routinely performing periodontal examinations.^6^

When accepting the challenges and difficulties of proper periodontal diagnosis, it should be remembered that other strategies (not for diagnosis) could be used. In this sense, one could propose different terms such as recognition, awareness, detection, screening, etc. could be used. For decades, Dentistry has searched for simplified periodontal diagnostic tools without success. However, the information given by this extensive work should not be disregarded. However, we repeat that diagnosis cannot be derived from these tools.

For example, it should be borne in mind that screening is the main aim of any type of partial examination, thus, if periodontal disease is found by means of this approach, complete periodontal charting is mandatory. Susin et al.^22^ tested 7 partial recording protocols based both on full mouth and in half mouth examinations and observed that all partial examination protocols underestimated the occurrence of periodontal disease. The best partial recording protocol found in this study was probing 3 sites per tooth (mesio-buccal, mid-buccal and disto-lingual). Nevertheless, this is still time consuming. The more sever the disease is, the worse partial recording for periodontal diagnosis will be. Therefore, the recommendation is that if an individual has periodontitis, full-mouth periodontal probing must be performed.

An alternative has been proposed for periodontal screening; that is the so called basic periodontal examination (periodontal screening and recording).^6^ This examination is based on probing all the teeth and scoring the sextant according to probing depth. When deeper probing depths are observed, a full-mouth periodontal examination is recommended. This is an interesting alternative, for those who understand that periodontal charting is not necessary. With this tool, it should be stressed that underestimation of periodontal diagnosis is a reality. On the other hand, if this were the only alternative considered for periodontal diagnosis, this would allow screening of more severe cases. Screening is an effective way of covering a larger number of the population. In different settings, this should be subject to discussion. The premise is that “doing something is better than doing nothing”. This is partially true, especially in individual situations. It should be re-emphasized that screening is not diagnosis.

Several studies have been conducted in the last years with the AAP/EFP classification system. These studies included epidemiological surveys and clinical studies. It should be borne in mind that it is only possible and feasible to use the AAP/EFP classification together with complete periodontal charting.^8,10^

In addition to interviewing the patient and performing periodontal physical examination, additional diagnostic tests are available. Image tests are the most used in terms of periodontal diagnosis. However, considering the international guidelines for radioprotection, they should be preceded by clinical indication, i.e. data from either the interview with the patient or from the physical examination are the core factors for indicating imaging examinations. The most common image tests used in periodontal diagnosis are periapical and panoramic radiographs, and more contemporarily, the cone-beam computed tomography (CBCT). All of them expose the individual to x-rays and therefore need to be limited. This article recommends that the practitioners must be aware of the international guidelines for radioprotection before indicating such examinations.^23^ The AAP/EFP classification system clearly uses information of past disease by means of image tests. This is important for standardizing periodontal diagnosis, however, as previously stated, should follow radioprotection guidelines.

The panoramic radiograph is one of the most cost-effective images, however, in cases of periodontal breakdown, it offers limited image detail. Therefore, in cases of moderate disease, complementation with selected periapical or vertical bitewing images is warranted, and in cases of severe periodontal disease, a complete periapical radiographic examination could be necessary. The use of CBCT is restricted to specific periodontal situations, including endo-perio relationships, fractures, perforations, etc.^24,25^

The most important aspect of image tests is that they are comprehensively analyzed, in order to yield a better diagnosis. In the specific case of periodontal diseases, the bone crest deserves special attention, both in terms of the presence of lamina dura (which might be indication of periodontal stability), and the amount of lost periodontal bone, especially for future analyses of disease progression. The advice is to exercise caution when emphasizing the presence of lamina dura, as its visibility can also be influenced by the angle of X-ray projection.

Sophisticated diagnostic methods have been proposed in the literature, including microbiological, immunological, physical, molecular assays.^26,27^ These methods have been extensively used in research. However, for the clinical approach, they have not proved to be necessary up to now. Whereas the desire is that more accurate diagnostic tools will be developed in the future, since the available tools are still based on probing, which is rudimentary and could be replaced by a more precise device. In terms of diagnosis of periodontal diseases, it should be borne in mind that the sophisticated tools are not and do not have to be routinely used.

Furthermore considering the complexity of periodontal disease, it is not possible to think of the diagnosis of periodontal diseases outside the concept of integral care, which includes additional tests (glycated hemoglobin data in diabetic individuals, or the aid of other blood tests in systemically compromised individuals); in addition to this, however, the professional should remember that to take care of a human being during the clinical examination since it is necessary to consider aspects such as: life history, family dynamics, exposure to risk factors, social aspects and psychology.

Contemporarily, self-report measurements of periodontal diseases, either combined with some clinical assessment, or not have been developed. They have been tested and validated against the gold standard, which is complete periodontal charting, in addition to the interview with the patient and additional imaging exams^28^ Although they might have been validated, they should not be used for diagnosis of periodontal diseases. These tools are not meant for diagnosis, but they shed some light in terms of increasing awareness, recognition of disease and referral to a dental professional. There are several examples with use of these types of tools . The Periodontal Risk Assessment (PRA)^29^ is a system that uses clinical data, together with information from the patient in order to establish risk and to suggest some clinical approaches. The Gingival Health Test is an internet-based questionnaire that is meant to increase awareness of periodontal diseases. However, it is not meant at all for diagnosis.^30^

### Goals for Latin America: concluding remarks

This section reviewed the key aspects of periodontal diagnosis, in an evidence-based approach, in an endeavor to summarize the state of the art, and taking into consideration the characteristics of the dental profession in Latin American countries. These countries have experienced continuous development in oral health care. The efforts in preventing and treating periodontal diseases still have not produced tangible effects in the region. The prevalence of periodontal diseases is still high, and a burden of disease is observed across Latin-American countries. In addition, differences in, cultural and socioeconomic characteristics are common in the area, which call for specific approaches. With the aim of increasing the quality of the profession even further, the following aspects should be considered:

- A call for action is necessary to increase awareness of periodontal diseases to enhance the quality of oral health care and proper maintenance of teeth throughout the patient’s life;
- Dental professionals should be trained right from the undergraduate curriculum to appropriately diagnose periodontal diseases and to successfully achieve prevention of periodontal diseases;
- Dental professionals need to increase awareness in the community about periodontal diseases. Therefore, the information that gingival bleeding is not normal, and that other characteristics of periodontal diseases such as tooth spacing or mobility might be signs of periodontal disease, etc. should be spread.

The use of self-reported periodontal awareness tools should be emphasized (based on the high prevalence of gingivitis and periodontitis in the Latin American community). It should be mandatory to emphasize periodontal diagnostic maneuvers regardless of the patient´s reason for consultation.

The definition that periodontal diagnosis needs complete periodontal charting needs to be spread. Other types of examination do no achieve diagnosis and are only useful for screening/recognition. Underdiagnosis needs to be avoided.

- Dental practitioners should be aware of systemic and behavioral aspects that are linked to periodontal diseases and include them in the interview with the patient; they must be able to work at a multidisciplinary level in cases in which this is required to accomplish overall health;
- Oral health professionals should routinely perform periodontal clinical examination, according to the level of disease of the patient;
- Additional diagnostic tests should be understood as being part of periodontal diagnosis and practitioners should know how to use them to obtain their best diagnostic yield;
- Dental professionals should understand the periodontal health/disease process to enable them to approach it correctly, either by themselves or to refer their patients to obtain a proper approach to treatment;
- The evolution of periodontal diagnosis and awareness needs to be continuously evaluated and under surveillance in the Latin American region, in order to increase the quality of practicing dentists.

Dental education needs to include more in depth periodontal diagnosis methodology(?) at all levels of healthcare.

## References

[1] Rösing CK, Cavagni J, Malheiros Z, Stewart B, Aránguis Freyhofer V (2020). Periodontal disease and its impact on general health in Latin America. Section IV: Diagnosis. Braz Oral Res.

[2] American Academy of Periodontology (2024). Glossary of periodontal terms.

[3] Cardoso EM, Reis C, Manzanares-Céspedes MC (2018). Chronic periodontitis, inflammatory cytokines, and interrelationship with other chronic diseases. Postgrad Med.

[4] Haag DG, Peres KG, Balasubramanian M, Brennan DS (2017). Oral conditions and health-related quality of life: a systematic review. J Dent Res.

[5] Sanz M, Ceriello A, Buysschaert M, Chapple I, Demmer RT, Graziani F (2018). Scientific evidence on the links between periodontal diseases and diabetes: consensus report and guidelines of the joint workshop on periodontal diseases and diabetes by the International Diabetes Federation and the European Federation of Periodontology. J Clin Periodontol.

[6] Preshaw PM (2015). Detection and diagnosis of periodontal conditions amenable to prevention. BMC Oral Health.

[7] Ke L, Nogueira G, Thomson WM (2023). Influence of case definitions on epidemiological estimates of periodontitis prevalence and its associations with smoking and OHRQoL. Community Dent Oral Epidemiol.

[8] Holtfreter B, Kuhr K, Borof K, Tonetti MS, Sanz M, Kornman K (2024). ACES: A new framework for the application of the 2018 periodontal status classification scheme to epidemiological survey data. J Clin Periodontol.

[9] Armitage GC (1999). Development of a classification system for periodontal diseases and conditions. Ann Periodontol.

[10] Caton JG, Armitage G, Berglundh T, Chapple IL, Jepsen S, Kornman KS (2018). A new classification scheme for periodontal and peri-implant diseases and conditions - Introduction and key changes from the 1999 classification. J Periodontol.

[11] Watt RG, Daly B, Allison P, Macpherson LM, Venturelli R, Listl S (2019). Ending the neglect of global oral health: time for radical action. Lancet.

[12] Hugo FN, Kassebaum NJ, Marcenes W, Bernabé E (2021). Role of dentistry in global health: challenges and research priorities. J Dent Res.

[13] Kopp SL, Ramseier CA, Ratka-Krüger P, Woelber JP (2017). Motivational interviewing as an adjunct to periodontal therapy-a systematic review. Front Psychol.

[14] Stenman J, Wennström JL, Abrahamsson KH (2018). A brief motivational interviewing as an adjunct to periodontal therapy-A potential tool to reduce relapse in oral hygiene behaviours: a three-year study. Int J Dent Hyg.

[15] Gillam DG, Yusuf H (2019). Brief motivational interviewing in dental practice. Dent J.

[16] Teles R, Moss K, Preisser JS, Genco R, Giannobile WV, Corby P (2018). Patterns of periodontal disease progression based on linear mixed models of clinical attachment loss. J Clin Periodontol.

[17] Donos N (2018). The periodontal pocket. Periodontol 2000.

[18] Medina-Vega M, Ibarra MC, Quezada-Conde MD, Reis IN, Frias AC, Raggio DP (2024). Periodontal status among 12-year-old schoolchildren: a population-based cross-sectional study in Quito, Ecuador. Braz Oral Res.

[19] Botero JE, Rösing CK, Duque A, Jaramillo A, Contreras A (2015). Periodontal disease in children and adolescents of Latin America. Periodontol 2000.

[20] Kingman A, Susin C, Albandar JM (2008). Effect of partial recording protocols on severity estimates of periodontal disease. J Clin Periodontol.

[21] Silva-Boghossian CM, Amaral CS, Maia LC, Luiz RR, Colombo AP (2008). Manual and electronic probing of the periodontal attachment level in untreated periodontitis: a systematic review. J Dent.

[22] Susin C, Kingman A, Albandar JM (2005). Effect of partial recording protocols on estimates of prevalence of periodontal disease. J Periodontol.

[23] Dula K, Benic GI, Bornstein M, Dagassan-Berndt D, Filippi A, Hicklin S (2015). SADMFR Guidelines for the use of cone-beam computed tomography/digital volume tomography. Swiss Dent J.

[24] Tugnait A, Carmichael F (2005). Use of radiographs in the diagnosis of periodontal disease. Dental update.

[25] Ismail A, Al Yafi F (2024). The role of radiographic imaging in the diagnosis and management of periodontal and peri-implant diseases. Dent Clin North Am.

[26] Armitage GC (2013). Learned and unlearned concepts in periodontal diagnostics: a 50-year perspective. Periodontol 2000.

[27] Kikuchi T, Hayashi JI, Mitani A (2022). Next-generation examination, diagnosis, and personalized medicine in periodontal disease. J Pers Med.

[28] Cyrino RM, Miranda Cota LO, Pereira Lages EJ, Bastos Lages EM, Costa FO (2011). Evaluation of self-reported measures for prediction of periodontitis in a sample of Brazilians. J Periodontol.

[29] Lang NP, Tonetti MS (2003). Periodontal risk assessment (PRA) for patients in supportive periodontal therapy (SPT). Oral Health Prev Dent.

[30] Duque A, Benítez-Silva CG, Rösing CK (2023). Development of an application and proposal of recommendations to increase awareness of periodontal diseases for patients: concepts, rationale, and use. J Int Acad Periodontol.

